# Supplementary material for: Genome-wide association study identifies common variants associated with breast cancer in South African Black women
Source: Nat Commun. 2025 Apr 14;16:3542. doi: 10.1038/s41467-025-58789-0 (PMC11997036; doi:10.1038/s41467-025-58789-0)
Supplement: Supplementary file 1 — Supplementary Information [file 41467_2025_58789_MOESM1_ESM.pdf]

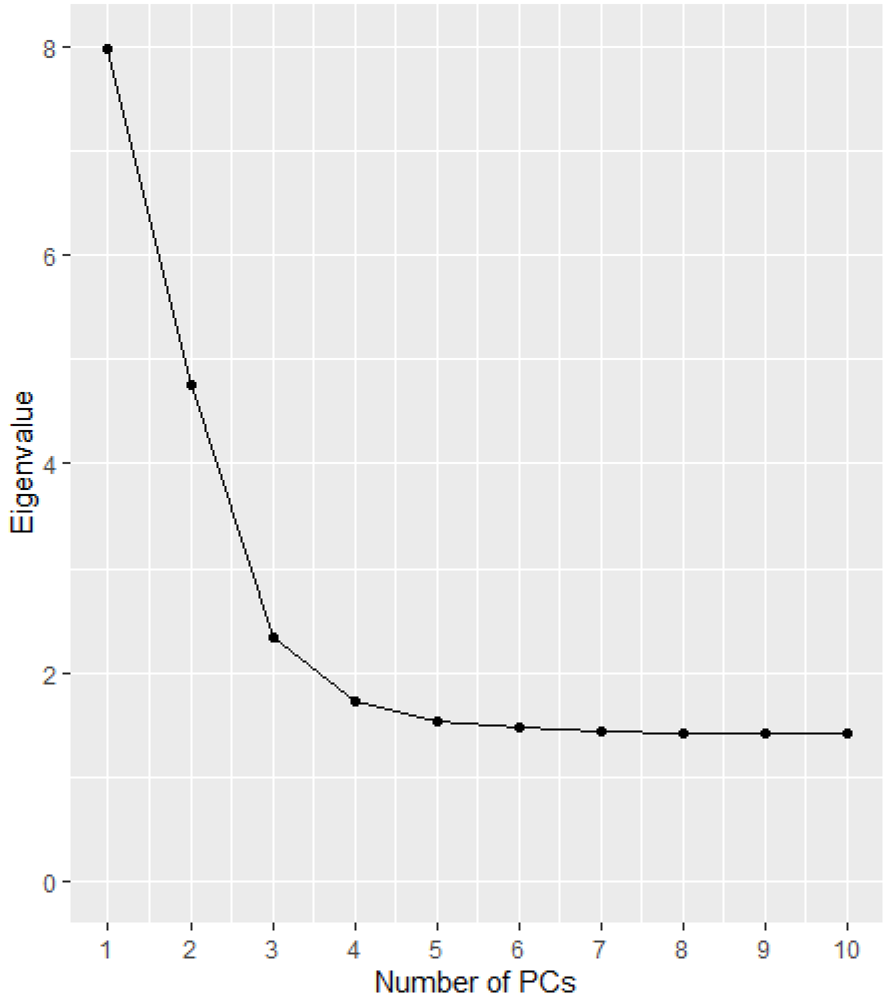

Figure S1: Eigenvalue curve showing that PCs 1-5 account for most of the variance observed.
(Principal components obtained using cases and controls after quality control)

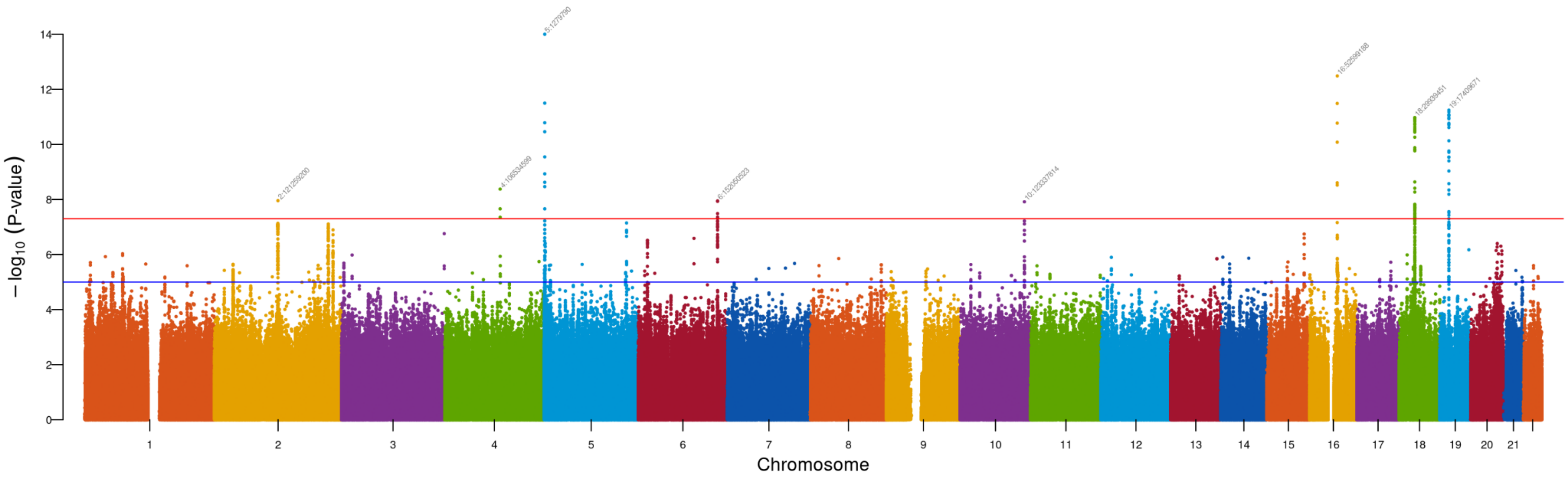

Figure S2: Manhattan plot of the meta-analysis of the African data from Jia *et al* (2024) and individuals of African Ancestry from the UK Biobank using METAL.

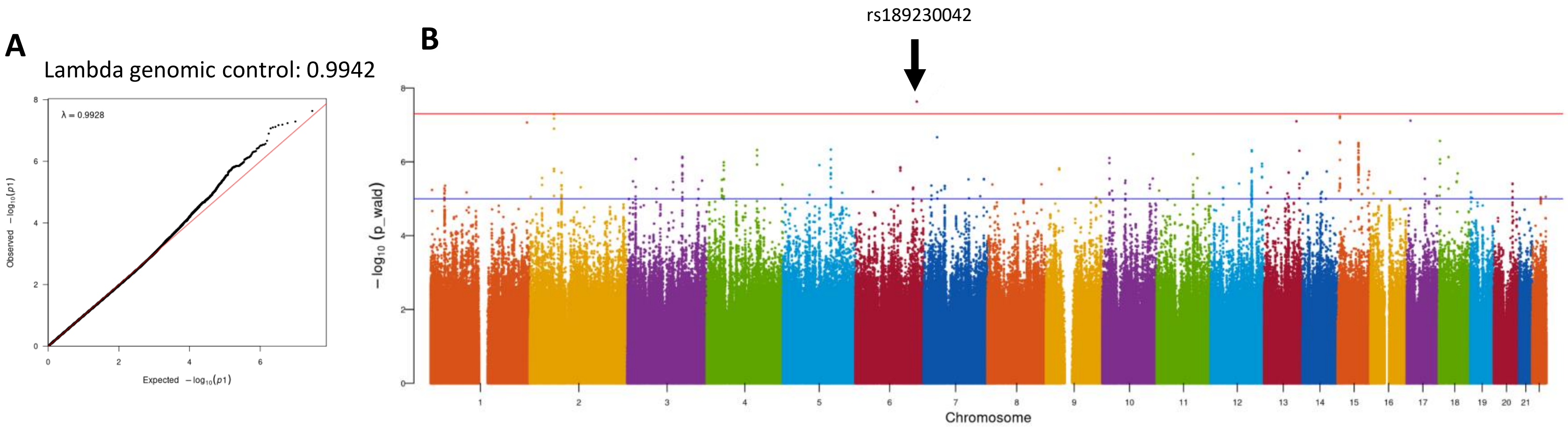

Figure S3: TNBC vs ER-positive BC, QQ-plot and Manhattan plot.

A. QQ plot, λ = 0.9942.

B. The Manhattan plot with genome-wide significant signal indicated on chromosome 6.

A

Lambda genomic control: 0.9972

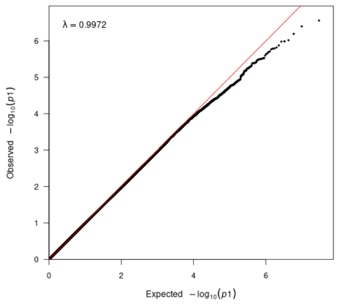

B

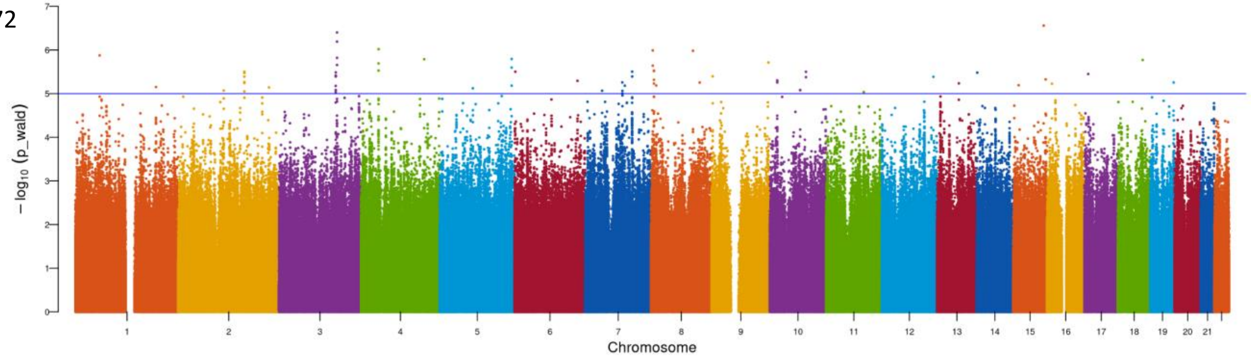

Figure S4: TNBC vs HER2-positive QQ-plot and Manhattan plot.

A. The QQ plot with lambda of 0.9972.

B. The Manhattan plot showing no genome-wide significant signals.

A B

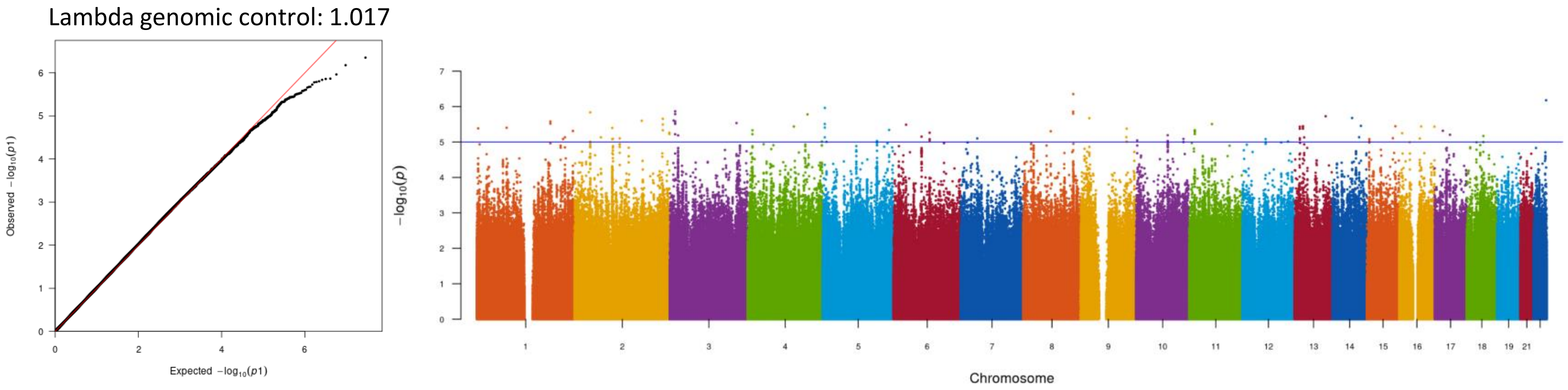

Figure S5: ER-positive vs controls QQ-plot and Manhattan plot

A. The QQ plot with lambda of 1.017.

B. The Manhattan plot showing no genome-wide significant signals.

ER = estrogen receptor, QQ quantile-quantile

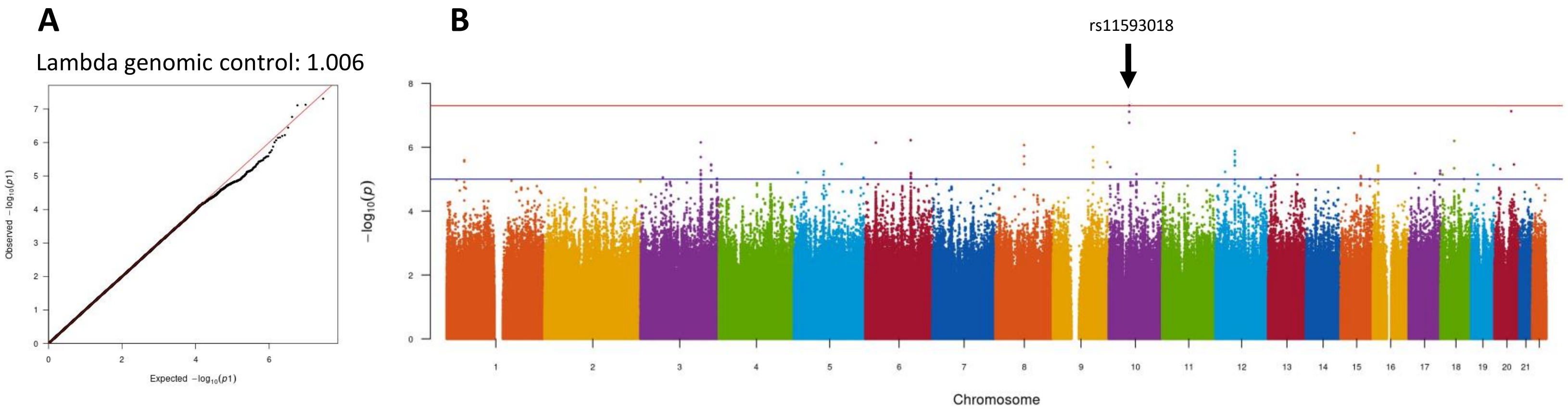

Figure S6: ER-negative vs control QQ-plot and Manhattan plot.

A. The QQ plot with lambda of 1.006

B. The Manhattan plot showing a signal on chromosome 10.

ER = estrogen receptor, QQ = quantile-quantile

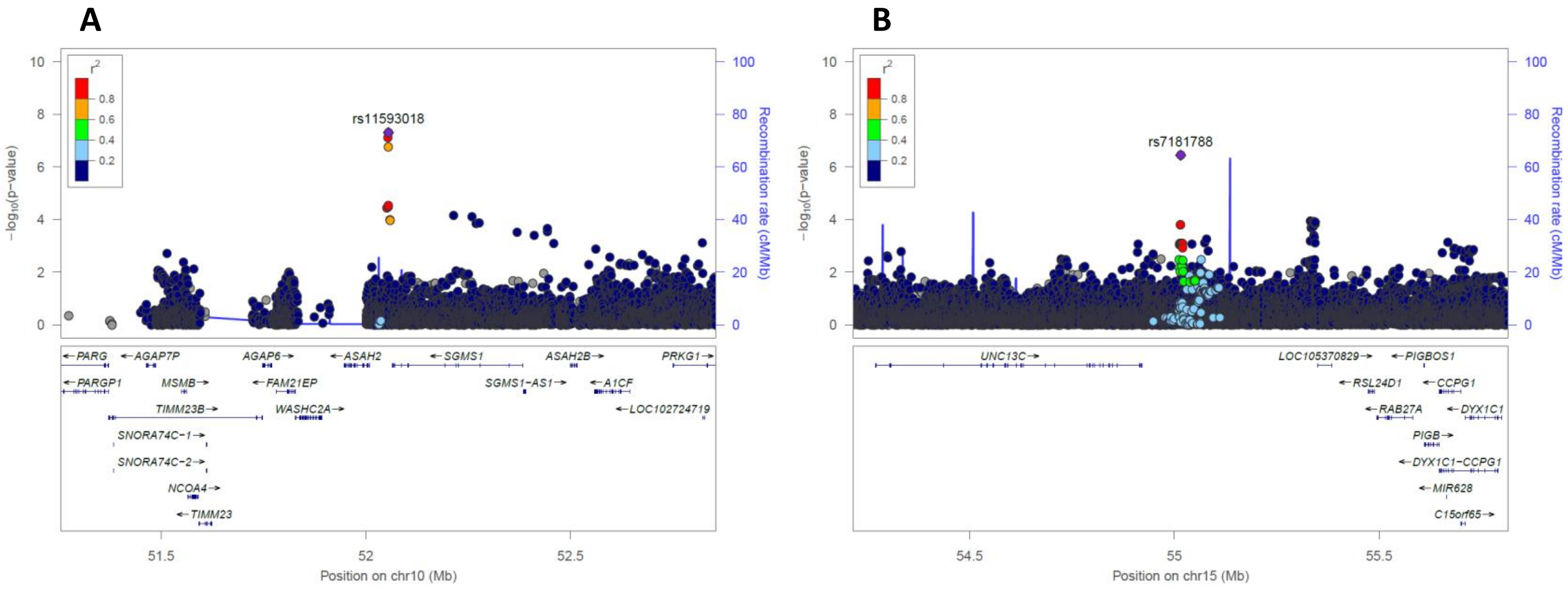

Figure S7: Regional association plots of the top signals in the ER-negative vs control analysis.
A. rs11593018 on chromosome 10.
B. r7181788 on chromosome 15.
ER = estrogen receptor

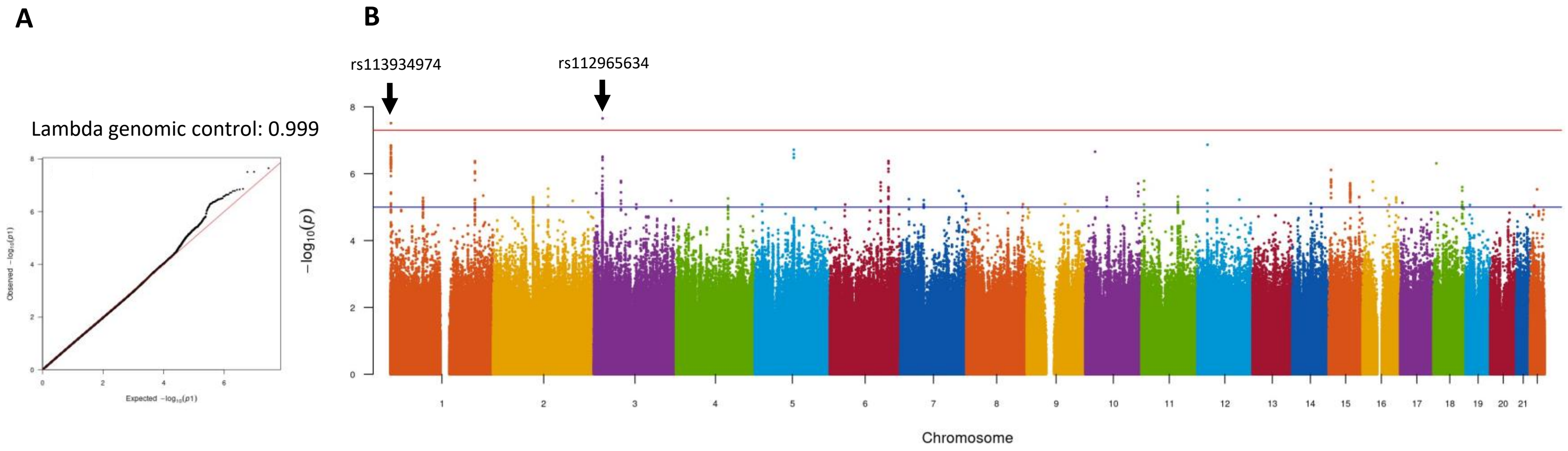

Figure S8: ER-positive vs ER-negative QQ-plot and Manhattan plot.

A. The QQ plot with lambda of 0.999.

B. The Manhattan plot showing signals on chromosomes 1 and 3.

ER = estrogen receptor, QQ = quantile-quantile

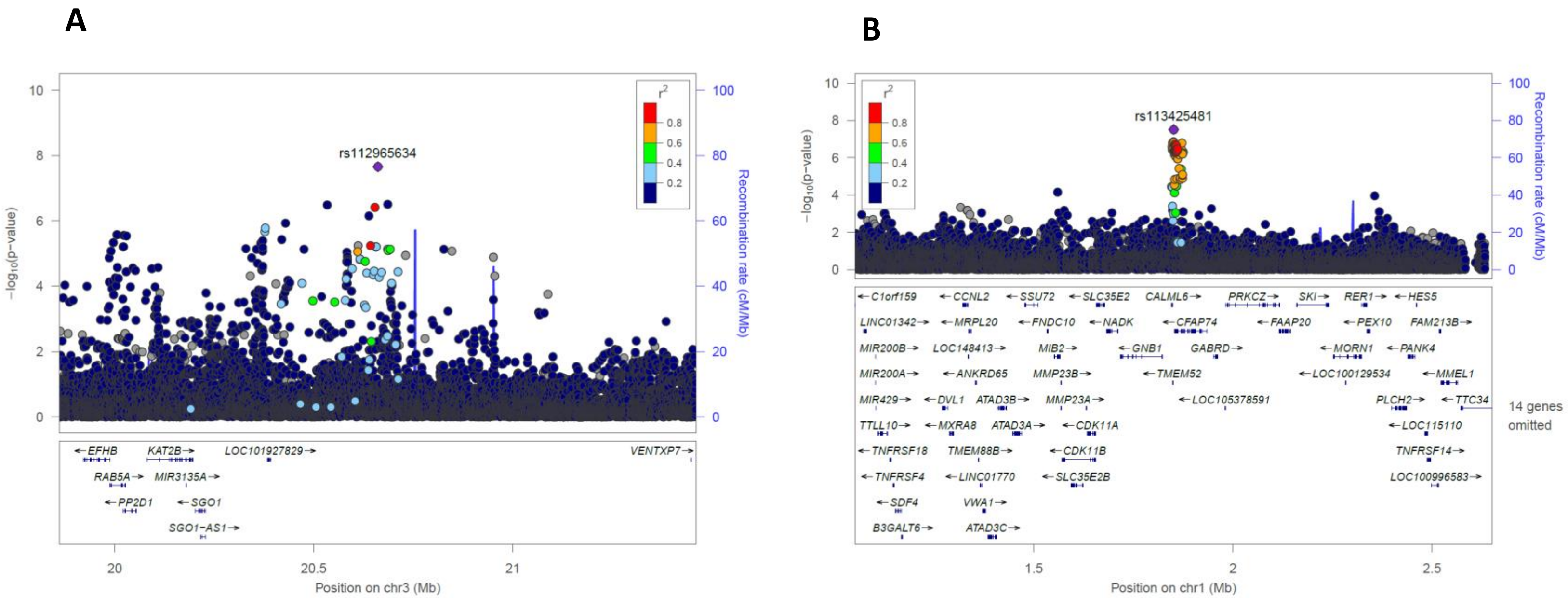

Figure S9: Regional association plots of the top signals in the ER-positive vs ER-negative analysis.

A. rs112965634 on chromosome 3.

B. r113425481 on chromosome 1.

ER = estrogen receptor
