# Supplementary material for: Genome-wide association study identifies common variants associated with breast cancer in South African Black women
Source: Nat Commun. 2025 Apr 14;16:3542. doi: 10.1038/s41467-025-58789-0 (PMC11997036; doi:10.1038/s41467-025-58789-0)
Supplement: Supplementary file 2 — Description of Additional Supplementary Files [file 41467_2025_58789_MOESM2_ESM.pdf]

Description of Additional Supplementary Files

File Name: supp_dataset_1
Description: Tables summarising the number of participants removed from the South African JCS dataset after admixture filters were applied and the number of participants included in the various analyses.

File Name: supp_dataset_2
Description: Lists the credible set of causal variants on chromosomes 15 and 17.

File Name: supp_dataset_3
Description: Lists the 89 additional SNPs with suggestive association (p < 5E-06) with breast cancer from the South African JCS GWAS.

File Name: supp_dataset_4
Description: Lists the results from the replication of the top 33 independent signals (p < 5E-06) from the South African JCS BC GWAS in the African Ancestry meta-analysis dataset.

File Name: supp_dataset_5
Description: Lists the replication of the top signals (p < 5E-06) from the African Ancestry meta-analysis in the South African JCS BC GWAS.

File Name: supp_dataset_6
Description: Lists the replication of the top signals (p<5E-06) from the South African JCS ER-negative/control analysis in the Jia et al dataset.

File Name: supp_dataset_7
Description: Lists the replication of the top signals (p<5E-06) from the ER-negative/controls from the Jia et al dataset in the South African JCS ER-negative analysis.

File Name: supp_dataset_8
Description: Lists the replication of the top signals (p<5E-06) from the ER-positive/controls Jia et al dataset in the South African JCS ER-positive dataset.
