# Supplementary material for: Genome-wide association study identifies common variants associated with breast cancer in South African Black women
Source: Nat Commun. 2025 Apr 14;16:3542. doi: 10.1038/s41467-025-58789-0 (PMC11997036; doi:10.1038/s41467-025-58789-0)
Supplement: Supplementary file 11 — Reporting Summary [file 41467_2025_58789_MOESM11_ESM.pdf]

nature portfolio

Corresponding author(s): Mahtaab Hayat

Last updated by author(s): Feb 27, 2025

Reporting Summary

Nature Portfolio wishes to improve the reproducibility of the work that we publish. This form provides structure for consistency and transparency in reporting. For further information on Nature Portfolio policies, see our Editorial Policies and the Editorial Policy Checklist.

Statistics

For all statistical analyses, confirm that the following items are present in the figure legend, table legend, main text, or Methods section.

n/a	Confirmed	
☐	☒	The exact sample size (*n*) for each experimental group/condition, given as a discrete number and unit of measurement
☒	☐	A statement on whether measurements were taken from distinct samples or whether the same sample was measured repeatedly
☐	☒	The statistical test(s) used AND whether they are one- or two-sided<br>*Only common tests should be described solely by name; describe more complex techniques in the Methods section.*
☐	☒	A description of all covariates tested
☐	☒	A description of any assumptions or corrections, such as tests of normality and adjustment for multiple comparisons
☐	☒	A full description of the statistical parameters including central tendency (e.g. means) or other basic estimates (e.g. regression coefficient) AND variation (e.g. standard deviation) or associated estimates of uncertainty (e.g. confidence intervals)
☐	☒	For null hypothesis testing, the test statistic (e.g. *F*, *t*, *r*) with confidence intervals, effect sizes, degrees of freedom and *P* value noted<br>*Give P values as exact values whenever suitable.*
☐	☒	For Bayesian analysis, information on the choice of priors and Markov chain Monte Carlo settings
☒	☐	For hierarchical and complex designs, identification of the appropriate level for tests and full reporting of outcomes
☐	☒	Estimates of effect sizes (e.g. Cohen's *d*, Pearson's *r*), indicating how they were calculated

Our web collection on statistics for biologists contains articles on many of the points above.

Software and code

Policy information about availability of computer code

Data collection	No software was used for data collection
Data analysis	METAL, Gemma v 0.98.1, PLINK v 1.9, STATA 18 and R v r4.3.3 and nextflow V1 were used

For manuscripts utilizing custom algorithms or software that are central to the research but not yet described in published literature, software must be made available to editors and reviewers. We strongly encourage code deposition in a community repository (e.g. GitHub). See the Nature Portfolio guidelines for submitting code & software for further information.

Data

Policy information about availability of data

All manuscripts must include a data availability statement. This statement should provide the following information, where applicable:

- Accession codes, unique identifiers, or web links for publicly available datasets
- A description of any restrictions on data availability
- For clinical datasets or third party data, please ensure that the statement adheres to our policy

The breast cancer data generated in this study have been deposited in the GWAS Catalog and will be available in the upon publication (GCST90551892, GCST90551893, GCST90551894, GCST90551895, GCST90551896, GCST90551897, GCST90551898). The breast cancer data used in this study are available to interested researchers through the European Genome-Phenome Archive (EGA), subject to controlled access review by the Data and Biospecimen Access Committee of the University of the Witwatersrand (BC cases and JCS controls accession number: EGAS00001008032). The AWI-Gen data used in this study are available to interested researchers through EGA, subject to controlled access review by the Data and Biospecimen Access Committee of the H3Africa Consortium; AWI-Gen

genotype dataset accession number: EGAD00010001996. The AWI-Gen data will be available for computational benchmarking studies on condition that no attempt is made to reidentify participants. Access to the dataset will require ethics approval from a recognized ethics committee.

Research involving human participants, their data, or biological material

Policy information about studies with human participants or human data. See also policy information about sex, gender (identity/presentation), and sexual orientation and race, ethnicity and racism.

Reporting on sex and gender	This study investigated breast cancer risk in females only. The sex was self-reported and then verified using genotype data
Reporting on race, ethnicity, or other socially relevant groupings	We used self-reported ethnicity as a proxy for ethnicity and was controlled for using principal components and admixture plots.
Population characteristics	The only covariates used in this study were principal components.
Recruitment	Participants were recruited from two studies: the Johannesburg Cancer Study where participants were recruited over 20 years, and the AWI-Gen study.
Ethics oversight	The protocol was approved by the WITS University Human Research Ethics Committee (M160807)

Note that full information on the approval of the study protocol must also be provided in the manuscript.

Field-specific reporting

Please select the one below that is the best fit for your research. If you are not sure, read the appropriate sections before making your selection.

☒ Life sciences ☐ Behavioural & social sciences ☐ Ecological, evolutionary & environmental sciences

For a reference copy of the document with all sections, see nature.com/documents/nr-reporting-summary-flat.pdf

Life sciences study design

All studies must disclose on these points even when the disclosure is negative.

Sample size	Samples with breast cancer were collected over a time period of 20 years. DNA was extracted from viable blood samples and these constituted the sample case numbers. The control size was determined by the available data from the AWI-Gen study. Sample size was limited by funding and accessibility to patients and controls.
Data exclusions	Data exclusions based on DNA quality and genotyping performance was done.
Replication	The findings from our paper were not replicated in populations with West African ancestry and this may be due to genetic heterogeneity between African populations, however, further validation of these results are needed.
Randomization	Randomisation is not required as cases are all samples with breast cancer, and controls are those without.
Blinding	Blinding is not required as we need to know who has breast cancer and who doesn't in order to determine differences in allele frequencies between the two groups. Laboratories did not have access to any phenotype data of samples being genotyped.

Reporting for specific materials, systems and methods

We require information from authors about some types of materials, experimental systems and methods used in many studies. Here, indicate whether each material, system or method listed is relevant to your study. If you are not sure if a list item applies to your research, read the appropriate section before selecting a response.

Materials & experimental systems

n/a	Involved in the study	
☒	☐	Antibodies
☒	☐	Eukaryotic cell lines
☒	☐	Palaeontology and archaeology
☒	☐	Animals and other organisms
☒	☐	Clinical data
☒	☐	Dual use research of concern
☒	☐	Plants

Methods

n/a	Involved in the study	
☒	☐	ChIP-seq
☒	☐	Flow cytometry
☒	☐	MRI-based neuroimaging

Plants

Seed stocks	*Report on the source of all seed stocks or other plant material used. If applicable, state the seed stock centre and catalogue number. If plant specimens were collected from the field, describe the collection location, date and sampling procedures.*
Novel plant genotypes	*Describe the methods by which all novel plant genotypes were produced. This includes those generated by transgenic approaches, gene editing, chemical/radiation-based mutagenesis and hybridization. For transgenic lines, describe the transformation method, the number of independent lines analyzed and the generation upon which experiments were performed. For gene-edited lines, describe the editor used, the endogenous sequence targeted for editing, the targeting guide RNA sequence (if applicable) and how the editor was applied.*
Authentication	*Describe any authentication procedures for each seed stock used or novel genotype generated. Describe any experiments used to assess the effect of a mutation and, where applicable, how potential secondary effects (e.g. second site T-DNA insertions, mosiacism, off-target gene editing) were examined.*
